# Microtensile Bond Strength of Self-Adhesive Luting Cements to Ceramics

**DOI:** 10.1155/2012/278623

**Published:** 2012-04-23

**Authors:** Tomoko Abo, Shigeru Uno, Masahiro Yoshiyama, Toshimoto Yamada, Nobuhiro Hanada

**Affiliations:** ^1^Department of Translational Research, Tsurumi University School of Dental Medicine, 2-1-3, Tsurumi, Tsurumi-ku, Yokohama 230-8501, Japan; ^2^Department of Dentistry, Toranomon Hospital, 2-2-2 Toranomon, Minato-ku, Tokyo 105-8570, Japan; ^3^Department of Operative Dentistry, Okayama University Graduate School of Medicine, Dentistry and Pharmaceutical Sciences, 2-5-1 Shikata-cho, Okayama 700-8525, Japan

## Abstract

The purpose of this paper was to compare the bond strengths of the self-adhesive luting cements between ceramics and resin cores and examine their relation to the cement thickness. Three self-adhesive luting cements (Smartcem, Maxcem, and G-CEM) and a resin cement (Panavia F 2.0) for control were used in the paper. The thickness of the cements was controlled in approximately 25, 50, 100, or 200 **μ**m. Each 10 specimens were made according to the manufacturers' instructions and stored in water at 37°C. After 24 hours, microtensile bond strength (**μ**TBS) was measured. There were significant differences in cements. Three self-adhesive cements showed significantly lower **μ**TBSs than control that required both etching and priming before cementation (Tukey, *P* < 0.05). The cement thickness of 50 or 100 **μ**m tended to induce the highest **μ**TBSs for each self-adhesive luting cements though no difference was found.

## 1. Introduction

Esthetic dentistry, including ceramic restorations, is now a great demand from the patients. CAD/CAM technology in dentistry has also become popular. One of the technologies, CEREC system, since its development in 1985, has improved the software and hardware for easier operation and better adaptation. The current CEREC 3 system can fabricate more precise inlays, onlays, crowns, and veneers. In a review on the CEREC restorations, Fasbinder summarized the postoperative sensitivity, restoration fracture, color match, margin adaptation, clinical longevity, and clinical performance [1]. However, the CAD/CAM system still has a problem with the fitting quality of the restorations. Mörmann and Schug compared the precision of fit between the CEREC 1 and CEREC 2 systems [2]. They reported that the mean marginal interface was 84 ± 38 *μ*m for CEREC 1-generated inlays and 56 ± 27 *μ*m for CEREC 2-generated inlays. Nakamura et al. reported a marginal gap of 53 to 67 *μ*m for CEREC 3-generated crowns [3].

Vitablocs Mark II (Vita Zahnfabrik, Germany), conventional feldspathic ceramic, is generally used in the CEREC system. The ceramic restorations are usually cemented with resin-based composite luting agent, after surface treatments necessary for the bonding. In the CEREC restoration, the luting material may be charged of two functions as a luting material and a restorative material to adhere between the tooth substrates and CEREC restoration with good mechanical properties and reliable bond capacity [4]. Therefore, the failure of the luting material at the margin may affect the longevity of restorations. In other words, proper selection of a luting agent is a last important decision in a series of steps that require meticulous execution and will determine the long-term success of fixed restorations [5].

Recently, newly developed resin luting cements called “self-adhesive luting cements” have been commercialized from several manufacturers. These materials feature that the adhesion is possibly achieved to various surfaces without surface pretreatment such as air-abrasion and/or HF-etching. However, there is little information on the performance of self-adhesive luting cements in the CEREC restorations without surface pretreatment.

*In vitro* bonding efficacy is often evaluated by measuring bond strength as well as morphological structures at the bonding interface. Therefore, the purpose of this study was to compare the bond strengths of the self-adhesive luting cements with different cement thickness, simulating the luting between ceramics and resin abutments without surface pretreatment.

## 2. Material and Methods

### 2.1. Specimen Preparation

**Figure 1:**
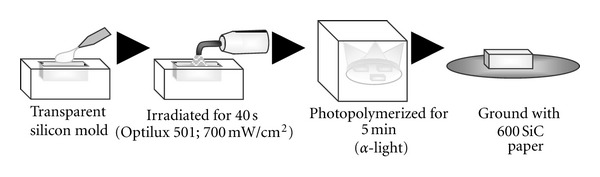
Schematic illustration of the procedure for core resin preparation.

Commercial 3 self-adhesive luting cements (Smartcem, Maxcem, and G-CEM) and a control cement (Panavia F 2.0) were used to bond two selected adherends, a ceramic block and resin core in this study (Table 1). Feldspathic ceramic blocks (Vitablocs Mark II; Vita Zahnfabrik, Germany) were horizontally cut with a low-speed diamond saw (Isomet; Buehler, Lake Bluff, IL, USA) and ground with #600 SiC paper to standardize the surface roughness. For preparation of the resin core blocks (Figure 1), core resin (Clearfil DC Core Automix; Kuraray Medical, Tokyo, Japan) was filled into a silicon mold (area: 8 × 10 mm^2^; height: 5 mm) as a bulk. The resin was irradiated from both opposing sides for 40 sec each with Optilux 501 (700 mW/cm^2^; SDS Kerr, Danbury, CT, USA), then post-cured for 5 min within a box of *α*-Light (Morita, Tokyo, Japan). The core resin blocks were ground with # 600 SiC paper after 24 h storage at 37°C.

### 2.2. Microtensile Bond Strength (*μ*TBS) Test

**Figure 2:**
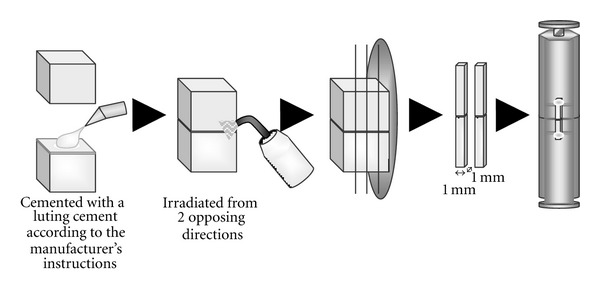
Schematic illustration of the procedure for *μ*TBS measurement.

The surface of the core resin block was covered with masking tapes (transparent tape with a circular hole, 6 mm in diameters) to standardize cement thickness: 25, 50, 100, and 200 *μ*m. A pilot study confirmed the thickness variation was ±1 *μ*m for each group. Three self-adhesive luting cements were mixed according to the manufacturers' instructions and filled into the hole of the tape without surface treatment (Table 2). Then, a ceramic block was put on it with mild finger pressure. Before cementation with Panavia F 2.0, both adherend blocks were etched with K-etchant Gel (Kuraray Medical, Tokyo, Japan) and silanated with the mixture of Clearfil SE primer (Kuraray Medical, Tokyo, Japan) and Clearfil Porcelain Bond Activator (Kuraray Medical, Tokyo, Japan) according to the manufacturer's instructions (Table 2). The cement was laterally irradiated from 2 opposing sides under each irradiation condition. The specimens were sectioned into 1.0 × 1.0 mm beams (*n* = 10 × 16 groups) after 24 h storage in water at 37°C. Individual beams were then attached to a Ciucchi's device [6] with cyanoacrylate glue (Model Repair II Blue; Dentsply-Sankin, Tochigi, Japan), and *μ*TBSs were measured using a universal testing machine (EZ Test; Shimadzu, Kyoto, Japan) at a crosshead speed of 1.0 mm/min (Figure 2). 

### 2.3. Failure Analysis

After measuring *μ*TBSs, the specimens were examined using Scanning Electron Microscope (SEM; DS-750, Topcon, Japan) to determine the failure modes. Failure modes were categorized as follows: adhesive failure at the interface between ceramic/core resin and cement, cohesive failure within cement, or mixed failure.

### 2.4. Statistical Analyses

The results of the *μ*TBS test were analyzed with two-way ANOVA with variables of cements and cement thickness. Multiple comparisons were performed with Tukey's HSD test. The statistical analyses were carried out at 5% level of significance.

## 3. Results

The means and standard deviations (SD) of *μ*TBSs were given in Table 3. Two-way ANOVA showed an interactive influence between the cements and cement thickness (*P* < 0.05). The multiple comparisons by Tukey's HDS test revealed significant differences between cements (*P* < 0.05).

Panavia F 2.0 gave the stable and higher *μ*TBSs than the other 3 cements regardless of the cement thicknesses (*P* < 0.05). In 3 self-adhesive luting cements, there was no significant difference in *μ*TBSs among cement thickness, while the highest *μ*TBS was to be given between 50 *μ*m (Smartcem and G-CEM) and 100 *μ*m (Maxcem) (Table 3).

SEM analysis revealed that fracture mode was dominantly cohesive failure in the cement regardless of the type of cement and cement thickness.

## 4. Discussion

In this study, adhesion between ceramics and core resin was examined, simulating the luting between CEREC restorations and resin abutments.

Mazzitelli et al. concluded that the predominance of acid-base reactions or radical polymerization might explain the different responses to substrate wetness and raise concerns regarding their universal application both on vital and pulpless teeth [7]. Also, *μ*TBSs is commonly affected by the properties of the adherends. Therefore, *μ*TBSs in this study were measured using uniform substrates as fundamental indexes to reduce the individual difference of the adherends. Also, the cement line was irradiated from 2 opposing sides after the cementation of two kinds of blocks because several self-etching resin cements were to be used in the dual-cure mode under optimal polymerization condition [8].

Ceramic surface is usually sandblasted or abraded with diamond bar, and/or etched (e.g., phosphoric acid or hydrofluoric acid) prior to silane treatment [9, 10]. However, for Panavia F 2.0, etching and priming were required before cementation, but hydrofluoric acid etching not always necessary for ceramics surface. In a usual clinical way, the pretreatment with phosphoric acid and saline-coupling agent before cementation is simple and effective [11]. Besides, newly developed self-adhesive luting cements are featured on the reducible treatment. Actually, one-step approach with self-adhesive luting cements seemed to be simpler and less technique-sensitive than the conventional resin cements. This study focused on the effect of cement thickness on the bond between core resins and ceramic surface. The bond strength is attributed to a lot of variables involved. The reduced factors might facilitate to understand the bond performance. Thus, the pretreatment with hydrofluoric acid was not carried out in this study. The further study would make clear the effect of surface pretreatment such as a hydrofluoric acid etching. Kamada et al. reported the dual-cured resin luting agents provided much higher early bond strength to ceramic blocks for CEREC than chemically cured resin luting agents and maintained durable bond strength even after 20,000 thermocycles [12]. In this study, all 4 materials were dual-cure luting cements. Three self-adhesive luting cements showed relatively lower *μ*TBSs than the control material, Panavia F 2.0. The surface pretreatment might be one of the reasons for the different bond performance between self-adhesive luting cements and control, Panavia F.

All self-adhesive luting cement used in the study contains phosphoric ester monomer. Besides, 4-MET is added in both Smartcem and G-CEM. These functional acidic monomers possibly contribute to the adhesion. Further, The dominant fracture mode, that is, cohesive failure within the cement regardless of the bland of the cements, indicates that tensile stress concentrated to the cement body rather than the bonding interfaces. This implies that the mechanical property of the resin matrix mainly contributes to the bonding performance of the cements.

Han et al. reported that the pH values of 3 self-adhesive luting cements, Smartcem, Maxcem, and G-CEM, were lower than 4 at 90 seconds after mixing; G-CEM was the lowest (pH 1.8) and Smartcem was the highest (pH 3.6) [13]. They also stated that the low pH might have an etching effect but an adverse influence on the adhesion if the low pH were left too long. Several self-etch cements tend to show high initial acidity and gradual rise of pH during setting [8]. In this study, Smartcem showed relatively lower *μ*TBSs than the others, and G-CEM showed slightly higher *μ*TBSs than Smartcem. These differences may be due to the etching effect by the different pH.

The results of the study also suggested that the thickness of cements affected the *μ*TBSs for all self-adhesive luting cement. Filler size and consistency of the luting composites affect the film thickness [14, 15]. Filler particle size in all 3 self-adhesive cements was less than 5 *μ*m. Two cements except Smartcem contain angular-shaped inorganic fillers [13]. The filler shape of Smartcem may be a powerful variable for the cement thickness though its diffusion in the resin matrix.

G-CEM contains UDMA as a cross-linking monomer, owing to a lower molecular weight and to the greater flexibility of the urethane linkage [16]. Maxcem is mainly composed of base monomers, UDMA, Bis-GMA, and TEGDMA. Asmussen and Peutzfeldt reported that varying the relative amounts of UDMA, Bis-GMA, and TEGDMA had a significant effect on the mechanical properties of the resin composition [16]. Therefore, it can be speculated that base monomers have a large influence on the *μ*TBSs of the different cement thicknesses. Moreover, the ratio of base monomers and functional acidic monomers could be associated with the mechanical properties of the cement.

Usually, there is a relatively large discrepancy between a CEREC restoration and cavity walls due to the accuracy of the optical impression and milling. The space must be filled with luting cement. Therefore, the varied bond strength by the cement thickness could be disadvantageous for the longevity of the restoration.

Further study should be carried out to investigate the between mechanical properties of the self-adhesive luting cements and their bonding capacity, and also longevity of the bonding.

## 5. Conclusion

Three self-adhesive luting cements showed lower *μ*TBSs than Panavia F 2.0 that required surface treatments for the bonding. There were significant differences between cements; Smartcem showed the lowest and Panavia F 2.0 the highest *μ*TBSs (Tukey's HDS, *P* < 0.05). Panavia F 2.0 gave the stable *μ*TBSs regardless of the cement thickness. The results suggested that the cement thickness might have an influence on *μ*TBSs, for the self-adhesive luting cements.

## Figures and Tables

**Table 1:** Composition of the commercial resin-based composite luting cement.

Product name (Shade)	Lot no.	Composition	Manufacturer
Smartcem (Natural)	R0707B1	Base Paste: HEMA, 4-MET, PEM-F, Initiator, Inhibitor, others Catalyst Paste: 1,3-Butanediol dimethacrylate, Sulfuric acid salt, Tertiary amine, Inhibitor, others	DENTSPLY-Sankin, Tochigi, Japan
Maxcem (Clear)	2855305	Base Paste: UDMA, Camphorquinone, Fluoroaluminosilicate glass, others Catalyst Paste: Bis-GMA, TEGDMA, Glycerophosphatedimethacrylate, Barium aluminoborosilicate glass, others	SDS Kerr, Orange, USA
G-CEM (A2)	0702061	Powder: Fluoroaluminosilicate glass, Initiator, Pigment Liquid: 4-MET, Phosphoric acid ester monomer, UDMA, Dimethacrylate, water, Silicon dioxide, Initiator, Inhibitor	GC, Tokyo, Japan
Panavia F 2.0 Paste (Brown)	0293AB, 0155AA	Paste A: MDP, Methacrylate monomer, Filler, Initiator Paste B: Methacrylate monomer, Filler, NaF, Initiator, Pigment	Kuraray Medical, Tokyo, Japan

HEMA: 2-hydroxyethyl methacrylate; 4-MET: 4-methacryloxyethyl trimellitate; PEM-F: 5-methacryloxyethyloxy cyclophosphazene monofluoride; UDMA: urethane dimethacrylate; Bis-GMA: bisphenol-A-glycidyl dimethacrylate; TEGDMA: triethyleneglycol dimethacrylate; MDP: 10-methacryloyloxydecyl dihydrogen phosphate.

Before cementation with Panavia F 2.0, specimens were etched with K-etchant and silanated with Clearfil SE primer and Porcelain Bond Activator.

**Table 2:** The procedures for each resin-based composite luting cement.

Smartcem	Maxcem	G-CEM	Panavia F 2.0
↓	↓	↓	↓
hand-mixed for 20 sec	auto-mixed	hand-mixed for 20 sec	$\left(\begin{array}{c} \text{etched}\;\text{for}\;5\;\text{sec}\\ \text{rinsed}\;\text{and}\;\text{dried}\\ \text{silanated}\;\text{for}\;5\;\text{sec} \end{array}\right)$
↓	↓	↓	↓
cemented and held for 2 min	cemented and held for 90 sec	cemented and held for 90 sec	hand-mixed for 20 sec
↓	↓	↓	↓
irradiated for 30 sec	irradiated for 20 sec	irradiated for 10 sec	cemented and held for 2 min
			↓
			irradiated for 20 sec

**Table 3:** Microtensile bond strength (MPa).

	Smartcem	Maxcem	G-CEM	Panavia F 2.0
25 *μ*m	15.38 (4.06)^a, b, c, d^	13.75 (5.91)^a, b, c, d^	12.53 (8.68)^a, b, c^	45.32 (8.72)^e^
50 *μ*m	17.85 (5.64)^b, c, d^	16.38 (6.17)^a, b, c, d^	22.60 (6.40)^d^	46.35 (7.76)^e^
100 *μ*m	9.55 (2.38)^a, b^	20.16 (1.90)^c, d^	16.98 (3.53)^a, b, c, d^	43.72 (6.16)^e^
200 *μ*m	8.70 (2.63)^a^	16.41 (3.88)^a, b, c, d^	13.72 (2.74)^a, b, c, d^	39.39 (9.21)^e^

Mean (SD). Same letters denote no significant difference (*P* > 0.05).
